# Comparison of ART outcome in patients with poor ovarian response according to POSEIDON criteria

**DOI:** 10.1038/s41598-022-22859-w

**Published:** 2022-10-21

**Authors:** Hyun Joo Lee, Hye Kyung Noh, Jong Kil Joo

**Affiliations:** 1 Department of Obstetrics and Gynecology, Biomedical Research Institute Pusan National University Hospital, Pusan National University School of Medicine, 179, Gudeok-Ro, Seo-Gu, Busan, 49241 Republic of Korea; 2 Mama Papa & Baby Hospital, Ulsan, Republic of Korea

**Keywords:** Biomarkers, Endocrinology, Health care, Medical research, Molecular medicine, Risk factors

## Abstract

The aim of this study is to evaluate whether the patient-oriented strategies encompassing individualized oocyte number (POSEIDON) criteria can reflect the prognosis of controlled ovarian stimulation (COS) by comparing the results of assisted reproductive technologies (ART) between four POSEIDON and normal responder (NR) groups. In total, 225 patients were included in this retrospective observational study. The patients underwent various COS protocols and in vitro fertilization or intracytoplasmic sperm injection, followed by fresh or frozen embryo transfer. Based on their clinical and demographic data, patients were divided into four groups according to the POSEIDON classification, and their ART outcomes were evaluated. Statistical analyses were performed using R version 4.0.5, and a p-value of < 0.05 was considered significant. The NR group had the highest number of total oocytes retrieved and total embryos obtained, as well as the best ART outcome in terms of clinical pregnancy rate ([CPR], 47.6%). The POSEIDON groups 1 and 2 had better COS and ART outcomes than groups 3 and 4 (CPR, 22.6%, 22.1%, 16.7%, and 4.8% in groups 1–4, respectively); the patients in group 3 were younger than those in group 2 by definition, but their CPR was lower than that of patients in group 2. When comparing young and old women with low anti-Müllerian hormone (AMH) levels, the younger group (POSEIDON group 2) had better COS and ART outcomes than their older counterparts, especially POSEIDON group 4. A binary logistic regression adjusted for body mass index (BMI) comparing the ART outcomes of patients that did not get pregnant in the POSEIDON groups compared to the NR group showed odds ratios (ORs) (95% confidence interval) of 2.938 (1.496–5.768), 3.376 (1.848–6.167), 6.801 (2.740–16.881), and 20.497 (8.284–50.713) for groups 1, 2, 3, and 4, respectively. Ovarian reserve still seems to outweigh patients’ age when predicting the ART outcomes of low-responder infertile women, as suggested by the results of POSEIDON groups 2 and 3. However, when there are no differences in ovarian reserve, as in POSEIDON groups 2 and 4, younger women with low AMH have a higher probability of pregnancy than their AMH-matched older counterparts.

## Introduction

The proportion of female patients with advanced age and/or low ovarian reserve visiting infertility clinics is increasing worldwide^1–3^. The estimated prevalence of poor ovarian response (POR) ranges from 6 to 35% according to various definitions and remains a scientifically controversial and clinically complex issue in the setting of assisted reproductive technology (ART); patients with POR usually cannot achieve the optimal number of oocytes after ovarian stimulation^4^. Common ovarian reserve biomarkers, such as anti-Müllerian hormone (AMH) and antral follicle count (AFC), are now widely used to predict the ovarian response to gonadotropin stimulation. However, these biomarkers are suboptimal for predicting reproductive success following ART because they are inadequate in identifying hyporesponder patients^3^. Hyporesponders are a subgroup of women with an impaired response to gonadotropin; therefore, a higher dose of gonadotropins or more prolonged stimulation is required to obtain an adequate number of oocytes. Therefore, hyporesponders should be distinguished from suboptimal or poor responders, who are simply classified by the number of oocytes retrieved^5^.

Although the Bologna criteria are currently the most widely used to identify patients with POR, their clinical use has been questioned because of the persistent heterogeneity among patients with POR^6^. Furthermore, the Bologna criteria cannot guide physicians in managing patients^7^. For example, according to the Bologna criteria, both young women with a low AMH and a previous episode of POR and older women with a normal AMH and a previous episode of POR would be included in the same “low prognosis” category, even though they require different clinical management according to their age or AMH levels.

To reflect ovarian resistance to gonadotropin stimulation (i.e., hyporesponsiveness), novel patient-oriented strategies encompassing individualized oocyte number (POSEIDON) criteria for patients with “low prognosis” have been conceptualized^8^. These criteria divide patients into four groups based on age, ovarian reserve markers such as AMH and/or AFC, and number of oocytes retrieved in a previous ovarian stimulation cycle. The POSEIDON criteria stratify women with POR into two main categories: the “unexpected POR” (groups 1 and 2) and “expected POR” (groups 3 and 4) groups. POSEIDON groups 1 (< 35 years old) and 2 (≥ 35 years old) were hypo-responders who unexpectedly showed a low ovarian response or resistance to ovarian stimulation with exogenous gonadotropins. These groups included women who had a poor (< 4) or suboptimal (4–9) number of oocytes retrieved after ovarian stimulation despite the presence of adequate ovarian reserve. POSEIDON groups 3 (< 35 years old) and 4 (≥ 35 years old) were expected to have a poor response to ovarian stimulation due to their low ovarian reserve; in particular, POSEIDON group 4 had a significantly poorer prognosis due to an age-related increase in oocyte aneuploidy^8^.

There have been only few reports comparing ART outcomes between the POSEIODON groups. In the current study, the authors classified patients with a low prognosis into four POSEIDON groups and patients without a low prognosis into the normal responder (NR) group. By comparing the results of ART between the POSEIDON and NR groups, the study evaluated whether the POSEIDON criteria greatly reflect the prognosis of controlled ovarian stimulation (COS), especially in Korean patients.

## Results

Table 1 shows the characteristics of all ART cycles. A total of 225 patients underwent one or more cycles, with the maximum number of cycles being five; 374 cycles were eligible for inclusion in the study. All POSEIDON and NR groups had a mean body mass index (BMI) < 25 kg/m^2^. The mean infertility duration was 36 months for POSEIDON groups 1 and 2, 24 months for POSEIDON groups 3 and NR, and 26 months for POSEIDON group 4. Primary infertility was more prevalent in groups 1 and 3 (75.5% and 86.1%), while groups 2 and 4 tended to have more patients with secondary infertility (57.1% and 60.7%). Female factor was the most common cause in groups 1, 2 and 4, whereas in group 3, most patients had unexplained cause. As in the classification, the age, AFC, and AMH were different between the POSEIDON groups. Cumulative gonadotropin consumption and duration of stimulation did not show statistical difference, along with the mean number of transferred embryos, among the study groups (data not shown).

**Table 1:** Patient characteristics in all performed cycles.

	Overall	NR	POSEIDON				*p*
			1	2	3	4	
No. cycles	374	124	53	77	36	84	
Age (yrs)^a^	35.47 (4.28)	33.59 (3.93)	32.32 (1.66)	37.56 (2.31)	31.97 (2.50)	39.81 (3.20)	0.000
No. prev. preg.^b^	0.00 [0.00, 8.00]	0.00 [0.00, 5.00]	0.00 [0.00, 5.00]	1.00 [0.00, 5.00]	0.00 [0.00, 5.00]	1.00 [0.00, 8.00]	0.000
HT (m)^a^	1.61 (0.05)	1.61 (0.05)	1.60 (0.05)	1.61 (0.05)	1.60 (0.06)	1.62 (0.05)	0.321
BW (kg)^a^	59.21 (10.62)	60.14 (10.70)	56.19 (8.24)	58.26 (8.79)	56.61 (15.74)	61.71 (10.09)	0.007
BMI (kg/m^2^)^a^	22.80 (3.89)	23.19 (3.93)	21.88 (3.40)	22.32 (3.01)	22.15 (6.45)	23.51 (3.23)	0.027
Infert. Dur. (mo)^b^	24.00 [1.00, 156.00]	24.00 [2.00, 144.00]	36.00 [8.00, 120.00]	36.00 [1.00, 156.00]	24.00 [5.00, 72.00]	26.00 [2.00, 144.00]	0.000
**Infert type (%)**^**c**^							
Prim	233 (62.3)	95 (76.6)	40 (75.5)	33 (42.9)	31 (86.1)	33 (39.3)	0.000
Sec	141 (37.7)	29 (23.4)	13 (24.5)	44 (57.1)	5 (13.9)	51 (60.7)	
**Infert factor (%)**^**c**^							
Male	67 (17.9)	33 (26.6)	15 (28.3)	9 (11.7)	2 (5.6)	8 (9.5)	0.000
Female	172 (46.0)	59 (47.6)	19 (35.8)	33 (42.9)	16 (44.4)	45 (53.6)	
Both	32 (8.6)	10 (8.1)	7 (13.2)	10 (13.0)	1 (2.8)	4 (4.8)	
Unexpl	103 (27.5)	22 (17.7)	12 (22.6)	25 (32.5)	17 (47.2)	27 (32.1)	
FSH^b^ (mIU/mL)	6.17 [0.11, 49.19]	5.01 [0.11, 14.20]	7.12 [1.20, 14.20]	6.35 [2.47, 34.64]	6.90 [1.84, 49.19]	7.74 [0.19, 30.60]	0.000
AMH^b^ (ng/mL)	2.08 [0.01, 23.94]	4.51 [1.44, 23.94]	2.87 [1.23, 12.31]	1.78 [0.77, 7.26]	0.31 [0.01, 4.15]	0.40 [0.02, 1.17]	0.000
AFC^b^	8.00 [0.00, 39.00]	12.00 [2.00, 39.00]	10.00 [0.00, 16.00]	8.00 [2.00, 18.00]	5.00 [2.00, 8.00]	3.00 [0.00, 10.00]	0.000
Preov. fol.^b^	10.00 [1.00, 76.00]	21.00 [10.00, 76.00]	10.00 [2.00, 18.00]	9.00 [1.00, 18.00]	5.00 [1.00, 21.00]	4.00 [1.00, 42.00]	0.000

Continuous variables are presented as either ^a^mean (SD) or ^b^median [interquartile range]. Categorical variables are presented as ^c^number of cycles (%). A p-value < 0.05 was considered statistically significant. *NR* normal responder, *No.* number, *yrs* years, *prev.* previous, *HT* height, *BW* body weight, *BMI* body mass index, *Infert*. Infertility, *Dur.* Duration, *mo* months, *prim.* Primary, *sec.* secondary, *unexpl.* Unexplained, *FSH* follicle stimulation hormone, *AMH* anti-Mullerian-hormone, *AFC* antral follicle count, *Preov. fol.* preovulatory follicles, *POSEIDON* patient-oriented strategies encompassing individualized oocyte number.

The ART outcomes among the four POSEIDON and NR groups are compared in Tables 2 and 3. The NR group had the highest number of total oocytes retrieved and total embryos obtained, calculated as a median (interquartile range) of 15.00 (10.00, 52.00) and 11.00 (3.00, 32.00), respectively. POSEIDON groups 1 and 2 had the higher numbers of oocytes retrieved and embryo obtained than groups 3 and 4. Overall, ET cycles up to 50% transferred blastocyst, 24.6% transferred morula, and the rest consisted 4-cell to 8-cell staged embryos (data not shown). Single ET was performed 40.6% of the entire patients, and the remaining 59.4% was double ET. The NR group had the best ART outcomes in terms of clinical pregnancy rate ([CPR], 47.6%), whereas the POSEIDON group 4 had the lowest CPR, regardless of the embryo stages and numbers (4.8%). POSEIDON groups 1 and 2 had significantly better pregnancy rates than groups 3 and 4 (CPR, 22.6%, 22.1%, 16.7%, and 4.8% for groups 1–4, respectively). Group 3 was younger than group 2 by definition, but their pregnancy outcome was lower than that of group 2.

**Table 2:** Comparison of ART outcomes in all performed cycles.

	Overall	NR	POSEIDON				*p*
			1	2	3	4	
No. cycles	374	124	53	77	36	84	
Total FSH per cycle^b^ (IU)	4800.00 [450.00, 25,950.00]	3862.50 [1425.00, 10,050.00]	4500.00 [1650.00, 9750.00]	5400.00 [2100.00, 11,250.00]	6300.00 [2250.00, 25,950.00]	5850.00 [450.00, 19,050.00]	0.000
Total oocytes retrieved^b^	6.00 [1.00, 52.00]	15.00 [10.00, 52.00]	5.00 [1.00, 11.00]	5.00 [1.00, 12.00]	2.00 [1.00, 14.00]	2.00 [1.00, 29.00]	0.000
GV^b^	0.00 [0.00, 8.00]	1.00 [0.00, 8.00]	0.00 [0.00, 4.00]	0.00 [0.00, 3.00]	0.00 [0.00, 2.00]	0.00 [0.00, 2.00]	0.000
M1^b^	0.00 [0.00, 6.00]	1.00 [0.00, 6.00]	0.00 [0.00, 5.00]	0.00 [0.00, 3.00]	1.00 [0.00, 3.00]	0.00 [0.00, 3.00]	0.000
M2^b^	4.00 [0.00, 39.00]	12.00 [0.00, 39.00]	4.00 [1.00, 9.00]	3.00 [0.00, 8.00]	2.00 [0.00, 12.00]	2.00 [0.00, 25.00]	0.000
Abnormal^b^	0.00 [0.00, 4.00]	0.00 [0.00, 3.00]	0.00 [0.00, 1.00]	0.00 [0.00, 2.00]	0.00 [0.00, 2.00]	0.00 [0.00, 4.00]	0.000
Total embryos^b^	4.00 [1.00, 32.00]	11.00 [3.00, 32.00]	4.00 [1.00, 9.00]	3.00 [1.00, 10.00]	2.00 [1.00, 11.00]	2.00 [1.00, 23.00]	0.000
Blastocyst^b^	0.00 [0.00, 13.00]	4.00 [0.00, 13.00]	0.00 [0.00, 6.00]	0.00 [0.00, 5.00]	0.00 [0.00, 6.00]	0.00 [0.00, 8.00]	0.000
Morula^a^	0.77 (1.62)	0.69 (2.12)	1.09 (1.67)	1.00 (1.48)	0.47 (0.70)	0.60 (1.02)	0.037
8-cell stage^a^	0.52 (1.01)	0.28 (1.08)	0.62 (1.21)	0.69 (0.98)	0.61 (0.73)	0.63 (0.83)	0.040
4-cell stage^b^	0.00 [0.00, 7.00]	0.00 [0.00, 7.00]	0.00 [0.00, 2.00]	0.00 [0.00, 3.00]	0.00 [0.00, 1.00]	0.00 [0.00, 2.00]	0.011
**Preg. (%)**^**c**^							
Chem. preg	35 (9.4)	17 (13.7)	7 (13.2)	8 (10.4)	1 (2.8)	2 (2.4)	0.000
Clinical preg	98 (26.2)	59 (47.6)	12 (22.6)	17 (22.1)	6 (16.7)	4 (4.8)	
No preg	241 (64.4)	48 (38.7)	34 (64.2)	52 (67.5)	29 (80.6)	78 (92.9)	

Continuous variables are presented as either ^a^mean (SD) or ^b^median [interquartile range]. Categorial variables are presented as ^c^number of cycles (%). A p-value < 0.05 was considered statistically significant. *NR* normal responder, *No.* number, *GV* germinal vesicle, *M1* metaphase I, *M2* metaphase II, *Preg.* Pregnancy, *Chem.* Chemical, *POSEIDON* patient-oriented strategies encompassing individualized oocyte number.

**Table 3:** Bonferroni correction p-value for multiple comparison of pregnancy rates (%).

1 vs 2	1 vs 3	1 vs 4	2 vs 3	2 vs 4	3 vs 4
1	1	0.0013*	1	0.0025*	0.9398

A p-value < 0.05 was considered statistically significant.

Table 4 describes the ART outcomes of no pregnancy among the POSEIDON groups compared with the NR group. A binary logistic regression adjusted for BMI showed odds ratios (ORs) (95% confidence interval) of 2.938 (1.496–5.768), 3.376 (1.848–6.167), 6.801 (2.740–16.881), and 20.497 (8.284–50.713) for groups 1, 2, 3, and 4, respectively.

**Table 4:** Binary logistic regression for ART outcomes of no pregnancy among POSEIDON groups compared to the NR group.

	Crude		Adjusted for BMI	
	OR [95% CI]	*p*	OR [95% CI]	*p*
NR	1 [Reference]		1 [Reference]	
POSEIDON				
1	2.833 [1.453, 5.524]	0.002	2.938 [1.496, 5.768]	0.002
2	3.293 [1.810, 5.991]	0	3.376 [1.848, 6.167]	0
3	6.560 [2.664, 16.150]	0	6.801 [2.740, 16.881]	0
4	20.583 [8.322, 50.910]	0	20.497 [8.284, 50.713]	0

Odds ratios are presented with 95% confidence interval. A p-value < 0.05 was considered statistically significant. *OR* odds ratio, *CI* confidence interval, *NR* normal responder, *BMI* body mass index, *POSEIDON* patient-oriented strategies encompassing individualized oocyte number, *ART* assisted reproductive technologies.

## Discussion

Assessing the prognosis of pregnancy before initiating ART is seemingly crucial for promoting patient compliance throughout the journey^9^. The concept “poor ovarian response” accounts for the quantity of oocytes of patients, mainly through the ovarian reserve; recently, it has been further enhanced by incorporating oocyte quality, as reflected by the patients’ age (5). Thus, poor ovarian responder patients could now be rethought as “low prognosis” patients, as stratified according to the POSEIDON criteria based on such quantitative and qualitative parameters and provided with an individualized approach^9^.

Numerous studies have evaluated and compared ART outcomes among POSEIDON groups with or without referring to the “non-POSEIDON” group, which is conventionally named the “normal ovarian responder” group, and there were varying results^9–15^. These studies were based on patients of different ethnicities, including Asian and Western countries; however, to the best of our knowledge, no study has evaluated Korean patients according to the POSEIDON classification. According to our study, POSEIDON groups 1 and 2, which were groups with normal ovarian reserves at any age, had significantly better pregnancy rates than groups 3 and 4 (CPR, 22.6%, 22.1%, 16.7%, and 4.8% for groups 1–4, respectively). Similarly, in a study by Efterkhar et al. on 245 Iranian patients with POR, POSEIDON groups 1 and 2 also had significantly higher pregnancy and live birth rates (LBR) than groups 3 and 4 (*p* < 0.05), as well as in an Indian study by Chinta et al.^13,16^. It could be postulated that ovarian reserve plays a more crucial role than age in deciding a patient’s ART prognosis; however, several Chinese studies have reported somewhat different results, stating higher pregnancy rates and LBRs in groups 1 and 3 compared to those in groups 2 and 4^14,17^. Particularly, Abdullah et al. observed that patients with POR with younger age and low prognosis (POSEIDON 3) showed an acceptable probability of live births and better perinatal outcomes compared to their older counterparts (POSEIDON 2), emphasizing the influence of age on ART prognosis and increasing the chances of conception with multiple ART cycles with proven evidence^17^. Moreover, according to the mathematically-validated model for predicting live birth rate after embryo transfer developed by Awadalla et al., age and embryo morphology take crucial component in deciding ART outcome^18,19^. The discrepancy of results between this and previous studies could have been due to the relatively lower AMH level in our patients, as the clinical nature of the current study was at a tertiary hospital specializing in treating older infertile patients with severely diminished ovarian reserve. The median (interquartile range) AMH levels (ng/mL) of groups 1, 2, 3, and 4 in the current study compared to those in Abdullah et al.’s study were as follows: 2.87 (1.23–12.31), 1.78 (0.77–7.26), 0.31 (0.01–1.14), and 0.40 (0.02–1.17) in the current study versus 5.7 (4.3–7.13), 3.12 (2.22–3.505), 1.2 (0.5–1.93), and 1.1 (0.22–1.31), respectively^17^. The age distributions of groups 1 and 2 in the current study are much lower than those in previous studies. The mean (standard deviation) age of the aforementioned groups was 32.32 (1.66) and 31.97 (2.50) in this study, respectively, as compared with the mean (interquartile range) ages of 28 (26–31) and 29 (28–30) in the study by Abdullah et al., respectively^17^. Thus, future studies including larger populations with more precisely sectioned ranges of patient age and AMH levels are required to deduce more concrete answers for ART outcomes according to the POSEIDON classification and their stratification reference for AMH levels. By evaluating such data and valuable previous literature through a comprehensive meta-analysis, more accurate prognosis development using the POSEIDON classification system may be possible.

However, despite the intertwined effect of ovarian reserve and oocyte quality on ART, when independently comparing the degrees of impact that serum AMH level and age exert on ART outcome expectations, AMH appears to be a more appropriate candidate according to the current study. When comparing only POSEIDON groups 2 and 3, group 2, which comprised older patients with a higher ovarian reserve, had higher pregnancy rates than group 3, yet there was no statistical significance after the BMI-adjusted Bonferroni correction for multiple comparisons (*p* = 1, clinical pregnancy rates of 22.1% for group 2 and 16.7% for group 3). Furthermore, the POSEIDON group 2 showed a significantly higher pregnancy rate than its age-matched lower-ovarian-reserve counterpart group 4 (*p* < 0.05, clinical pregnancy rates of 22.1% in group 2 and 4.8% in group 4). It has been well established that an AMH as low as 1.2 mg/mL is significantly correlated with poor ovarian response, especially in older patients^1,15,20^. Nevertheless, Tokura et al. reported relative contraception probability among patients with POR over 40 years of age with AMH levels < 0.4 ng/mL^20^. Detailed counselling needs to be offered to these aged women for the risks, success rates, and ethical and legal implications of these fertility treatment options; instead of warning them for possibly negative ART outcomes, older women should be screened and adjusted for underlying medical conditions that could have an impact on maternal and neonatal morbidity and mortality^21^.

This study has some limitations. The types of ovarian stimulation protocols are heterogeneous among patients, and they could not be manipulated because of the study’s retrospective nature. In this study, the most suitable stimulation protocols were decided by a specialized reproductive endocrinologist and embryologists under tertiary hospital conditions according to extensive experience and previous studies. The main focus of the study was not on the protocol itself, as infertile patients with low prognosis need to be provided with individualized ART treatments^22,23^. Similarly, statistical analysis was performed using combined patient cohorts of fresh and frozen embryo transfer (ET) procedures. The most important limitations of the current study are its small sample size and lack of live birth rate (LBR) data for each group. Because of the small number of patients, POSEIDON groups 1 and 2 could not be further categorized into subgroups to maintain the statistical significance of the analysis. However, as mentioned earlier, to our knowledge, this study is the first to apply the POSEIDON classification system to patients with POR of homogenous Korean ethnicity at a single, highly professional tertiary infertility clinic. Despite these limitations, the results of the current study can establish a basis to treat infertility in patients with POR. Further studies with a greater number of patients with POR, ART cycles, and LBR data to enhance the statistical significance and possibly the practical interpretations of the POSEIDON classification system in a real-world setting are warranted.

## Conclusions

After evaluation according to the POSEIDON classification system, the ovarian reserve could still outweigh the patient’s age when predicting the ART outcome of a low-responder infertile woman. However, when ovarian reserve is not statistically different between patients, younger women with a low prognosis have a higher probability of pregnancy than their AMH-matched older counterparts. Younger women, even those with a low prognosis, can still have a chance to increase their probability of ART success by undergoing multiple cycles of in vitro fertilization (IVF) or intracytoplasmic sperm injection (ICSI). Such a convenient, evidence-based prognosis decision process would be more successful with international scholarly efforts to examine the POSEIDON classification system in a larger number of women with POR with more precisely stratified ages and AMH levels.

## Methods

This study aimed to evaluate whether the POSEIDON criteria reflect the prognosis of COS by comparing the results of ART between the four POSEIDON groups and NR group. The requirement of approval by the Institutional Review Board (IRB) of the Pusan National University Hospital was waived owing to its retrospective observational nature, as well as its analysis of previously recorded and anonymized patient medical charts, therefore confirming that all methods were carried out in accordance with relevant guidelines and regulations. Nevertheless, all patients had been routinely explained with possibility of their clinical data being utilized for research purposes and had agreed with corresponding informed consent on their first visit to the center.

### Patient characteristics

This study was a retrospective, observational study, including 225 patients with 374 COS cycles who attended the Infertility Centre of Pusan National University Hospital from January 2017 to December 2020. Patient data were reviewed using the hospital’s electronic medical records system. Inclusion criteria were female patients who completed initial infertility work-ups and consequent COS cycles followed by fresh or frozen ET with several outpatient follow-ups for confirming clinical pregnancies. Clinical and demographic data were collected for all patients: age at COS, gravida, height, weight, BMI, duration and type of infertility and accompanying cause, AFC, serum follicle-stimulating hormone (FSH), and AMH. The COS protocol, dose, and duration of gonadotropin stimulation were determined individually according to the patients’ age, BMI, serum AMH level, AFC, and previous outcome of COS by a single professional infertility and reproductive endocrinologist. Exclusion criteria were patients who received or provided ovum donation, underwent fertility preservation process such as oocyte or embryo freezing, became aged to be included in another group, had severe male factors, abnormal natural killer cell levels or activity and/or thrombophilia, represented with uterine anomaly or synechiae, reported to smoke or drink, and/or had experienced unilateral oophorectomy or chemotherapy.

### Classification of patient groups

Patients without any low prognosis were classified into the NR group, and those with low prognosis were classified into the POSEIDON group and further divided into four main groups, as described below, according to previous literature:

Group 1: Patients aged < 35 years with sufficient ovarian reserve parameters (AMH ≥ 1.2 ng/mL) and an unexpected suboptimal/poor ovarian response.

Group 2: Patients aged ≥ 35 years with sufficient ovarian reserve parameters (AMH ≥ 1.2 ng/mL) and an unexpected suboptimal/poor ovarian response.

Group 3: Patients aged < 35 years with poor ovarian response parameters (AMH < 1.2 ng/mL).

Group 4: Patients aged ≥ 35 years with poor ovarian response parameters (AMH < 1.2 ng/mL)^8^.

### COS, oocyte retrieval, and ET

All patients underwent either a gonadotropin-releasing hormone (GnRH) agonist long protocol or GnRH antagonist protocol, followed by IVF or ICSI, with medical injections including recombinant follicle-stimulating hormone (rFSH), human menopausal gonadotropins (HMG), and/or either GnRH agonist or GnRH antagonist, as previously established^24^. Patients started with rFSH (Gonal-F, Merck, Serono, Switzerland) at an initial dose ranging from 75 to 300 IU and/or HMG (Menopur, Ferring pharmaceuticals, NJ, USA) at an initial dose ranging from 75 to 150 IU, for stimulation days from 10 to 16 days, until the final oocyte maturation. During COS, all patients were closely evaluated with serial transvaginal ultrasonographic measurements of follicle growth and endometrium to optimize the dosage of rFSH and HMG (Voluson E6 General Electric, Milwaukee, Wauwatosa, WI, USA). Human chorionic gonadotropin (hCG) and/or GnRH agonist were administered when the dominant follicle reached ≥ 17 mm for final oocyte maturation. Oocytes were retrieved 35 h after trigger administration using ultrasonography-guided aspiration^25^. For all patients, no more than 2 cleavage embryos were transferred, and the same ET policy was applied to both fresh and frozen embryo transfers. Fresh embryo transfer was considered for the patients with low risk of ovarian hyperstimulation syndrome (OHSS), implantation failure and early pregnancy loss. When fresh ET was decided, it was performed 3 days after oocyte retrieval, and frozen ET was performed using appropriate endometrial preparations, as previously described^26^.

### Outcomes

The primary outcome was CPR, defined as the subsequent visualization of one or more gestational sacs of appropriate size to the serum β-hCG level and calculated gestational weeks using transvaginal ultrasonography after ET. The secondary outcomes included cases with no pregnancy, defined as having a serum β-hCG level < 5.0 mIU/mL measured at two weeks after the day of oocyte retrieval for fresh ET or on the 14th day from embryo fertilization for frozen ET or having failure to achieve 20 mIU/mL. Chemical pregnancy was defined as achieving serum β-hCG level ≥ 20 mIU/mL measured two weeks after the day of oocyte retrieval for fresh ET or on the 14th day from embryo fertilization for frozen ET but no gestational sac in subsequent weeks.

### Statistical analysis

All statistical analyses performed in this study were professionally discussed and consulted by the Department of Biostatistics, Biomedical Research Institute, Pusan National University Hospital. All statistical data were organized into a computerized database. Variables were evaluated for clinical significance using Fisher’s exact test for categorical variables and a one-way ANOVA or Kruskal–Wallis test after a normality test for continuous variables, where appropriate. Multiple comparisons were performed using a post-hoc analysis, and the Bonferroni method was used to correct the significance level. When analyzing ART outcomes of no pregnancy among the POSEIDON groups with reference to normal responders, a binary logistic regression model was used to perform univariate and multivariate analyses adjusted for BMI. R version 4.0.5 was employed in the analysis process, and a p-value < 0.05 was considered statistically significant.

### Ethical approval and consent to participate

The requirement of approval by the Institutional Review Board (IRB) of the Pusan National University Hospital was waived owing to its retrospective observational nature, as well as its analysis of previously recorded patient medical charts not containing any information allowing personal identification.

## Data Availability

The datasets generated and/or analyzed during the current study are not publicly available because their availability is only permitted when they are processed for research purposes and will be destroyed as soon as the analysis is completed. In some restricted cases, the datasets are available from the corresponding author with permission from the Pusan National University Hospital IRB upon reasonable request.
